# Nitrate ammonification by *Nautilia profundicola* AmH: experimental evidence consistent with a free hydroxylamine intermediate

**DOI:** 10.3389/fmicb.2013.00180

**Published:** 2013-07-04

**Authors:** Thomas E. Hanson, Barbara J. Campbell, Katie M. Kalis, Mark A. Campbell, Martin G. Klotz

**Affiliations:** ^1^School of Marine Science and Policy, University of DelawareNewark, DE, USA; ^2^Delaware Biotechnology Institute, University of DelawareNewark, DE, USA; ^3^Department of Biological Sciences, Clemson UniversityClemson, SC, USA; ^4^Department of Biology, University of North CarolinaCharlotte, NC, USA

**Keywords:** hydroxylamine, hydroxylamine oxidoreductase, nitrite, *Epsilonproteobacteria*, nitrate ammonification

## Abstract

The process of nitrate reduction via nitrite controls the fate and bioavailability of mineral nitrogen within ecosystems; i.e., whether it is retained as ammonium (ammonification) or lost as nitrous oxide or dinitrogen (denitrification). Here, we present experimental evidence for a novel pathway of microbial nitrate reduction, the reverse hydroxylamine:ubiquinone reductase module (reverse-HURM) pathway. Instead of a classical ammonia-forming nitrite reductase that performs a 6 electron-transfer process, the pathway is thought to employ two catalytic redox modules operating in sequence: the reverse-HURM reducing nitrite to hydroxylamine followed by a hydroxylamine reductase that converts hydroxylamine to ammonium. Experiments were performed on *Nautilia profundicola* strain AmH, whose genome sequence led to the reverse-HURM pathway proposal. *N. profundicola* produced ammonium from nitrate, which was assimilated into biomass. Furthermore, genes encoding the catalysts of the reverse-HURM pathway were preferentially expressed during growth of *N. profundicola* on nitrate as an electron acceptor relative to cultures grown on polysulfide as an electron acceptor. Finally, nitrate-grown cells of *N. profundicola* were able to rapidly and stoichiometrically convert high concentrations of hydroxylamine to ammonium in resting cell assays. These experiments are consistent with the reverse-HURM pathway and a free hydroxylamine intermediate, but could not definitively exclude direct nitrite reduction to ammonium by the reverse-HURM with hydroxylamine as an off-pathway product. *N. profundicola* and related organisms are models for a new pathway of nitrate ammonification that may have global impact due to the wide distribution of these organisms in hypoxic environments and symbiotic or pathogenic associations with animal hosts.

## Introduction

Nitrate is a major fixed nitrogen pool in the global N-cycle (Falkowski and Godfrey, 2008; Klotz and Stein, 2008). Nitrate can be reduced via denitrification to nitrous oxide or dinitrogen gas, whereby it is lost from terrestrial and aquatic ecosystems, or via ammonification to ammonium that can be retained (Brandes et al., 2007; Klotz and Stein, 2008, 2010). In both pathways, nitrate is reduced to nitrite. In heterotrophic denitrifiers and ammonia oxidizers, nitrite is further reduced by NO-forming nitrite reductases: NirS or NirK. Nitrite reduction to ammonium is catalyzed by assimilatory (NirB/NirA) or respiratory (NrfA) ammonia-forming nitrite reductase (Klotz and Stein, 2008, 2010). Ammonia-oxidizing chemolithotrophs and methanotrophs can also form NO by the oxidation of hydroxylamine, another potential route of nitrogen loss in terrestrial and aquatic ecosystems (Campbell et al., 2011). Several *Epsilonproteobacteria* that are able to use nitrate for respiration and/or as a sole source of nitrogen for growth do not encode homologs of either nitrite reductase type, suggesting that these organisms possess an unrecognized mechanism for nitrite reduction to ammonium.

**Figure 1:**
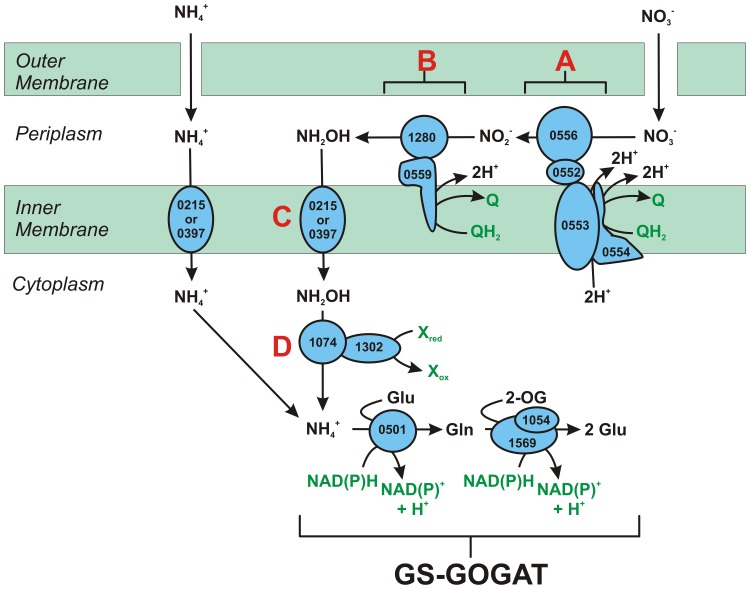
**The reverse-HURM pathway in *N. profundicola***. Individual steps are noted with capital letters in the figure. Numbers in the depicted proteins refer to locus tags from the AmH genome (i.e., 1280 = NAMH_1280 = HaoA). **(A)** Nitrate reduction by respiratory periplasmic nitrate reductase (NapABGH, 0556-53), **(B)** HURM (Hydroxylamine:Ubiquinone Redox Module) comprised of a reversely operating hydroxylamine oxidoreductase (HaoA, 1280) functioning as an ocataheme nitrite reductase, that produces hydroxylamine utilizing electrons donated from a tetra-heme cytochrome c in the NapC/NrfH/*c*_M_552 family (CycB, 0559). **(C)** Transport of hydroxylamine via ammonia transporters related to AmtB. **(D)** Reduction of hydroxylamine to ammonium by a hybrid cluster protein/hydroxylamine reductase (Hcp/Har, 1074) utilizing reducing power from a predicted Fe-S containing protein (1302) whose electron donor (X) is currently unknown. Assimilation of ammonium into biomass occurs via glutamine synthetase and glutamine:2-oxoglutarate aminotransferase (GS-GOGAT).

Genome annotation indicates that not only *Nautilia profundicola*, but several pathogenic *Campylobacter* spp. among the *Epsilonproteobacteria* may utilize nitrate as a nitrogen source for growth and/or as a terminal electron acceptor. *N. profundicola* is a member of the deepest branching lineage of the *Epsilonproteobacteria* (Campbell et al., 2006; Smith et al., 2008) and its relatives have only been found in deep-sea hydrothermal vent environments. In contrast, *Campylobacter* spp. are typically associated with animal hosts and their presence in other environments is ill defined. While the genomes of *N. profundicola, Campylobacter concisus, C. curvus*, and *C. fetus* contain genes encoding a periplasmic molybdopterin guanine dinucleotide-linked nitrate reductase complex (NAP), there are no genes encoding either ammonium- or NO-forming nitrite reductases. In addition, a *napC/cycB* gene was identified in the genome of *N. profundicola* (Campbell et al., 2009), which is unusual as the NAP complex in *Epsilonproteobacteria* usually lacks a NapC protein (Kern and Simon, 2009; Klotz and Stein, 2010; Simon and Klotz, 2013). Based on genome-informed metabolic reconstruction (Campbell et al., 2009), we recently proposed a novel pathway for nitrate assimilation (Figure 1) whereby nitrite is reduced to hydroxylamine by a hydroxylamine:ubiquinone redox module (HURM, i.e., a quinone-dependent hydroxylamine dehydrogenase), which we term the reverse-HURM pathway. In *N. profundicola*, the HURM is thought to consist of a NapC/NrfH-related cytochrome *c*_M_552 (*cycB*, NAMH_0559) that mediates electron transfer between the quinone pool and a periplasmic hydroxylamine oxidoreductase (HAO, NAMH_1280). HURM, operating in the forward direction and consisting of hydroxylamine dehydrogenase (EC:1.7.2.6; HaoA_3_) connected to a cytochrome *c* protein electron shuttle (*c*554 and/or *c*_M_552 encoded by *cycA* and *cycB*, respectively), links the oxidation of hydroxylamine to the quinone pool in aerobic ammonia-oxidizing bacteria (Klotz and Stein, 2008; Simon and Klotz, 2013). In anaerobic ammonia-oxidizing (anammox) bacteria, the redox module consists of hydrazine oxidoreductase and an as yet unidentified cytochrome *c* (Jetten et al., 2009; Simon and Klotz, 2013). In the reverse-HURM pathway, hydroxylamine generated from nitrite in the periplasm is transported into the cytoplasm and reduced to ammonium via a hydroxylamine reductase, also known as the hybrid cluster protein (Har/Hcp). Thus, the proposed electron accepting reactions of the reverse-HURM pathway are:

**Table:** 

	Reaction	Enzyme
(1)	2H^+^ + NO^−^_3_ + 2e^−^ → NO^−^_2_ + H_2_O	Nitrate reductase
(2)	5H^+^ + NO^−^_2_ + 4e^−^ → NH_2_OH + H_2_O	HURM
(3)	3H^+^ + NH_2_OH + 2e^−^ → NH_4+_ + H_2_O	Hydroxylamine reductase
(4)	10H^+^ + NO^−^_3_ + 8e^−^ → NH_4+_ + 3H_2_O	Sum

The genomes of *N. profundicola, C. concisus, C. curvus, C. fetus*, and *Caminibacter mediatlanticus* each contain genes encoding the enzymes of the reverse-HURM pathway while the genomes of other *Epsilonproteobacteria* encode homologues of the classical NO-forming NirS/NirK and ammonium-forming assimilatory siroheme NirA, or respiratory pentaheme NrfA nitrite reductases [Table 1, (Kern and Simon, 2009)]. We hypothesize that the reverse-HURM pathway improves the survival and/or dispersal of *N. profundicola* and *Campylobacter* spp. in ammonium-deficient host or non-host associated environments. However, for this hypothesis to be true, the reverse-HURM pathway must provide ammonium for biosynthesis and/or facilitate energy conservation in these organisms.

**Table 1:**
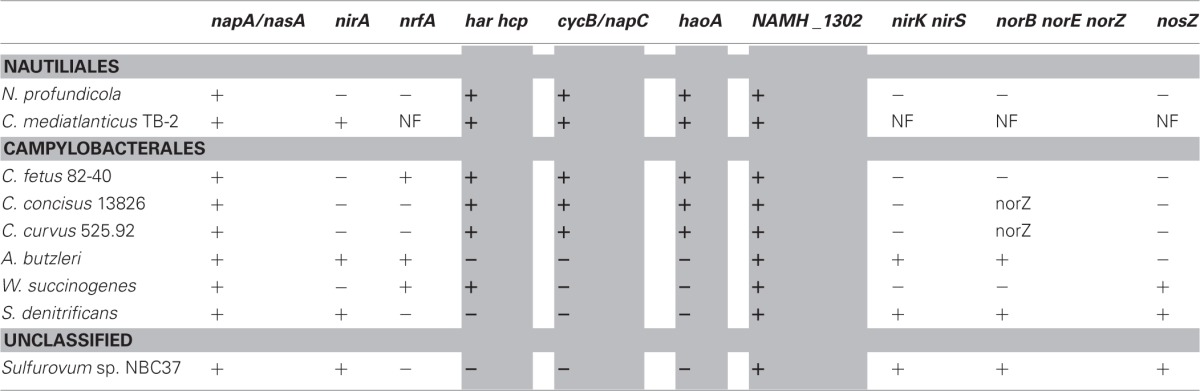
**Nitrogen metabolism gene inventories in genome-sequenced *Epsilonproteobacteria***.

Components of the proposed reverse-HURM pathway are noted in bold text in the shaded columns.

+, present in complete genome sequence; −, absent in complete genome sequence; NF, absent in draft genome sequence with multiple contigs.

## Results

### Energetics of growth in *N. profundicola*

*N. profundicola* utilizes both formate and hydrogen as electron donors for energy metabolism and is typically grown on polysulfide as the electron acceptor (Campbell et al., 2001; Smith et al., 2008). Calculations under standard conditions (1 M of all species, 25°C, pH = 7) indicate that when nitrate is reduced by the reactions of the reverse-HURM pathway, the Gibbs free energy (Δ G°) is substantially greater than that available from the reduction of polysulfide (Table 2). For example, the Δ G° per mole of formate oxidized with nitrate is 7.4-fold increased over polysulfide (−237 kJ mol^−1^ vs. −32 kJ mol^−1^). From this calculation, one can predict that the growth of *N. profundicola* should be stimulated in cultures with nitrate as the electron acceptor relative to polysulfide.

**Table 2:** **Calculated free energies of reaction under standard conditions (1 M all species, 25°C and pH = 7) for electron accepting half reactions of nitrogen compounds and polysulfide and coupled reactions with formate or hydrogen as the electron donor**.

	**E^0^ (V)**	**ΔG^0^ (kJ mol^−1^)**
**HALF REACTIONS**		
2H^+^ + NO^−^_3_ + 2e^−^ → NO^−^_2_ + H_2_O	+0.873	−169
5H^+^ + NO^−^_2_ + 4e^−^ → NH_2_OH + H_2_O	+0.270	−104
3H^+^ + NH_2_OH + 2e^−^ → NH_4+_ + H_2_O	+1.780	−344
S^2−^_3−8_ + H^+^ + 2e^−^ → S^2−^_2−7_ + HS^−^	−0.267	+52
	**Δ G^0^ (kJ mol^−1^)**	**per H_2_or CH_2_O_2_**
**COUPLED REACTIONS**		
H_2_ oxidation		
4H_2_ + NO^−^_3_ + 2H^+^ → NH_4+_ + 3 H_2_O	−937	−234
3H_2_ + NO^−^_2_ + 2H^+^ → NH_4+_ + 2 H_2_O	−688	−229
H_2_ + S^2−^_3−8_ → H^+^ + S^2−^_2−7_ + HS^−^	−28	−28
Formate oxidation		
4CH_2_O_2_ + NO^−^_3_ + 2H^+^ → 4CO_2_ + NH_4+_ + 3 H_2_O	−946	−237
3CH_2_O_2_ + NO^−^_2_ + 2H^+^ → 3CO_2_ + NH_4+_ + 2 H_2_O	−695	−232
CH_2_O_2_ + S^2−^_3−8_ → CO_2_ + S^2−^_2−7_ + H^+^ + HS^−^	−32	−32

### Growth of *N. profundicola* with nitrate

**Figure 2:**
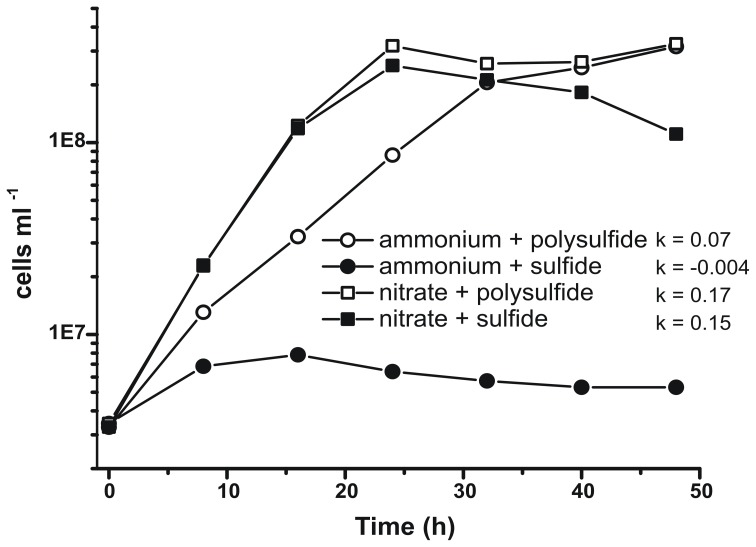
**Growth of *N. profundicola* with indicated electron acceptors, nitrogen compounds, and sulfur sources**. Growth rates were determined between 8 and 24 h; *k* = specific growth rate constant (h^−1^). Each point is the average of 2 or 3 independent biological replicates.

Prior characterization and growth of *N. profundicola* utilized media with polysulfide as a combined sulfur source and electron acceptor (Campbell et al., 2001, 2009; Smith et al., 2008). To further clarify the role of nitrate in the energy metabolism of *N. profundicola*, strain AmH was grown with sulfide as the sole sulfur source so that nitrate was the sole electron acceptor (Figure 2). The electron donor was a mixture of hydrogen and formate in a medium with no other sources of sulfur or nitrogen (Campbell et al., 2001). Growth rates with nitrate as potential electron acceptor and nitrogen source and sulfide (sulfur source) or polysulfide (sulfur source and potential electron acceptor) were more than double the growth rates observed in cultures using ammonium as the sole nitrogen source and polysulfide as an electron acceptor and sulfur source. As expected, negative control (no electron acceptor) cultures provided with ammonium as the nitrogen source and sulfide as the sulfur source were unable to grow.

### Nitrogen balance in *N. profundicola* cultures

**Figure 3:**
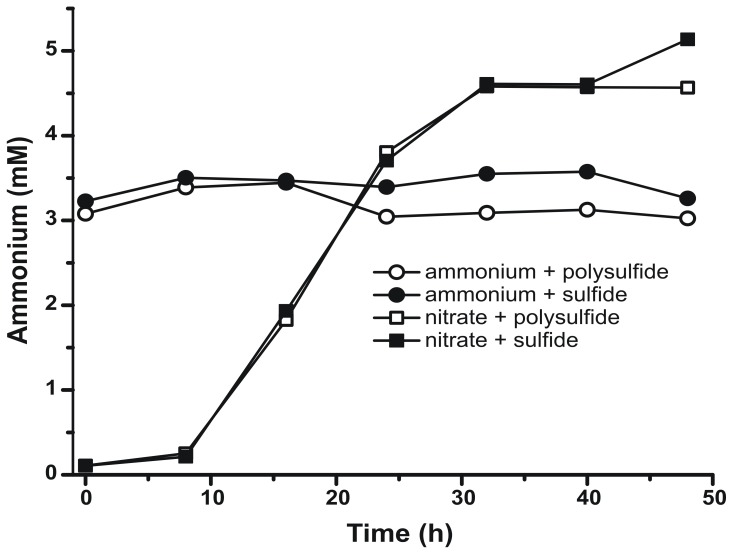
**Ammonium concentrations measured in cultures of *N. profundicola*-containing the indicated electron acceptors, nitrogen compounds, and sulfur sources**. Each point is the average of 2 or 3 independent biological replicates.

To confirm that the level of ammonium produced by *N. profundicola* was in excess of biosynthetic needs, we calculated the biosynthetic nitrogen demand as follows. Assuming a C:N ratio of 32:6.4 for exponentially growing bacterioplankton (Vrede et al., 2002), 288 fg of C μm^−3^ for exponentially growing *Escherichia coli* (Loferer-Krossbacher et al., 1998), and a cell volume of 0.021 μm^3^ calculated from the reported dimensions of *N. profundicola* (Smith et al., 2008), the production of 1 × 10^8^ cells ml^−1^ requires ~120 ng ml^−1^ of N (~9 μM), a figure that would not change regardless of the N-source utilized. *N. profundicola* did not utilize a significant amount of the ammonium provided during growth with polysulfide as the electron acceptor (Figure 3, open circles), which agrees with the low N requirement calculated to produce the observed cell densities. The ammonium plus sulfide culture did not grow (Figure 2) and also did not detectably consume ammonium (Figure 3, closed circles). In contrast, the amount of ammonium produced by cells grown with 5 mM nitrate was 4.6 mM with polysulfide and 5.2 mM with sulfide (Figure 3, open and closed squares, respectively). If the reverse-HURM pathway in the *Epsilonproteobacteria* primarily serves to produce ammonium for biosynthesis, we would expect low levels of exogenous ammonium to strongly repress HURM pathway gene expression and nitrate reduction activity. However, the stoichiometric conversion of nitrate to ammonium in excess of the predicted N requirement and observed ammonium consumption in the ammonium + polysulfide cultures is consistent with the hypothesis that nitrate is utilized as a terminal electron acceptor by *N. profundicola* and that nitrate ammonification is not subject to repression by ammonium.

### ^15^N-NO^−^_3_ incorporation into biomass

**Figure 4:**
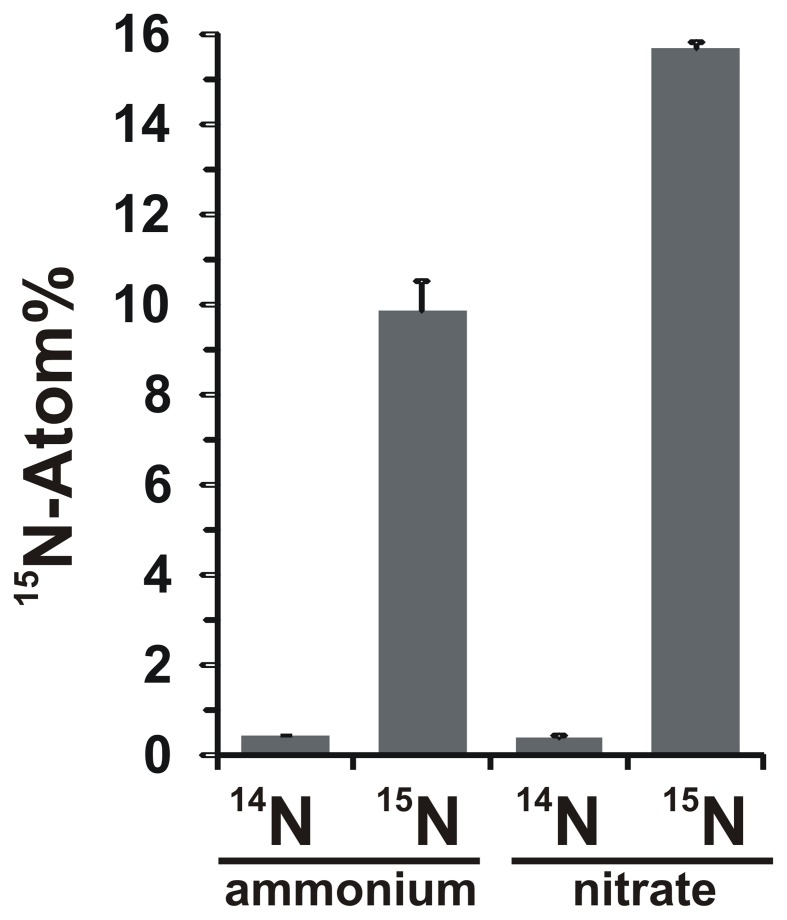
**Nitrogen uptake into *N. profundicola* biomass determined following one round of batch culture with 5 mM of the indicated nitrogen compounds supplied at natural abundance (^14^N) or at 20 Atom% ^15^N**. Values are averages of 3 independent biological replicates ± the standard deviation.

The potential for nitrate assimilation was assessed by examining the incorporation of ^15^N-labeled ammonium or nitrate into *N. profundicola* biomass during growth with all above combinations of electron donors and potential acceptors. *N. profundicola* grown on 20 Atom% ^15^N-labeled ammonium with polysulfide produced biomass enriched to ~10 Atom% ^15^N after one round of batch culturing (Figure 4), whereas the unlabeled control was not enriched in ^15^N. Cultures grown with sulfide and 20 Atom% 15N-labeled nitrate produced biomass enriched to 16 Atom% ^15^N (Figure 4). Nitrate-grown biomass contained approximately 5-fold more N than ammonium-grown biomass (data not shown), which may explain the more extensive enrichment of ^15^N in the nitrate-grown samples.

### Transcription of key pathway genes

**Figure 5:**
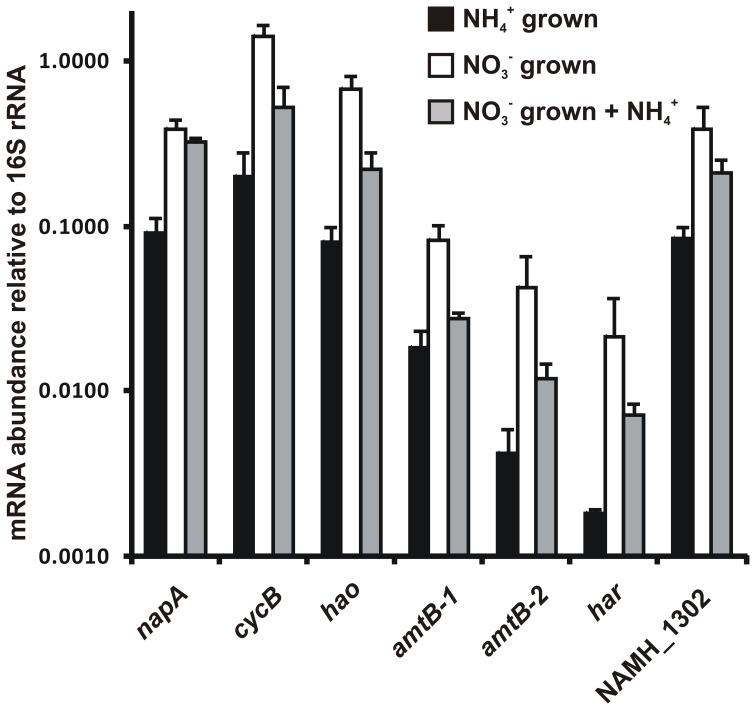
**Steady state mRNA levels for key genes encoding enzymes of the reverse-HURM pathway in *N. profundicola* grown with ammonium + polysulfide (black bars), nitrate + sulfide (white bars), or nitrate + polysulfide after the addition of ammonium**. Transcript abundance was determined by fluorescent RT-PCR and normalized to 16S rRNA abundance determined in each sample. Values are averages of 3 independent biological replicates ± the standard deviation.

Transcript abundance for genes that encode enzymes of the proposed reverse-HURM pathway in *N. profundicola* was determined by quantitative real-time PCR in batch cultures grown with ammonium or nitrate. All components of the proposed pathway displayed a strong increase in transcript abundance in nitrate grown cultures (Figure 5). mRNAs encoding a nitrate reductase subunit (*napA*) were 4.6-fold (*P* < 0.05, two-tailed heteroscedastic *t*-test) more abundant in nitrate + sulfide grown cells relative to ammonium + polysulfide grown cells. Transcripts of both components of the HURM, *haoA* and *cycB/napC*, were increased by 8.5- and 7.1-fold (*P* < 0.05 for both), respectively. Consistent with our prediction that hydroxylamine produced in the periplasm by the HURM would be transported through major facilitator protein channels, mRNA's encoding both homologs of the ammonium/methylammonium transporter *amtB* were increased by 4.5-fold (*amtB*-1, NAMH_0397, *P* < 0.08) and 10.3-fold (*amtB*-2, NAMH_0215, *P* > 0.10). Hydroxylamine is a powerful mutagen that must be detoxified as it arrives in the cytoplasm. We propose that this is accomplished by NADH-dependent Har/Hcp based on studies of the *hcp* gene in the nitrate assimilation gene cluster of *Rhodobacter capsulatus* E1F1 where it is required for the reduction and assimilation of hydroxylamine produced by a cytoplasmic assimilatory nitrate reductase (Cabello et al., 2004; Pino et al., 2006). Consistent with this proposal, the largest change in transcript abundance was seen for *har*, which increased by 11.7-fold, but this change was not significant (*P* > 0.10) due to the high variability in independent measurements of *har* transcript abundance. Originally, we had proposed that the electron donor to Har was an NADH dehydrogenase encoded by NAMH_0542 (Campbell et al., 2009). However, we now believe a better candidate for this function is NAMH_1302, a predicted 4Fe-4S cluster protein, based on conservation in other *Epsilonproteobacteria* (Table 1). Consistent with this prediction, transcripts of NAMH_1302 were 4.6-fold more abundant in nitrate grown cells relative to ammonium, but the change was not significant (*P* > 0.10).

To test for potential ammonium repression of pathway expression, ammonium was added to nitrate + sulfide grown cultures and the mRNA abundance quantified for the same genes. In general, ammonium addition decreased mRNA abundances of the reverse-HURM pathway encoding genes from 1.2- to 3.6-fold (Figure 5, gray bars), though this effect was only significant (*P* < 0.10) for *haoA* and *napC*/*cycB* transcripts that encode the HURM subunits. Interestingly, the expression of *napA* decreased the least (1.2-fold), indicating the need for nitrate respiration in these cells. In no case, did the addition of ammonium reduce transcript levels to those seen in cultures grown in ammonium + polysulfide.

### Evidence for a hydroxylamine intermediate

Direct measurements of hydroxylamine in filtrates of *N. profundicola* cultures grown with nitrate found low, but consistently detectable levels of hydroxylamine (5.7 ± 3.5 μM, *SD, n* = 3). This low level is consistent with an intermediate whose production and consumption rates are closely matched.

**Figure 6:**
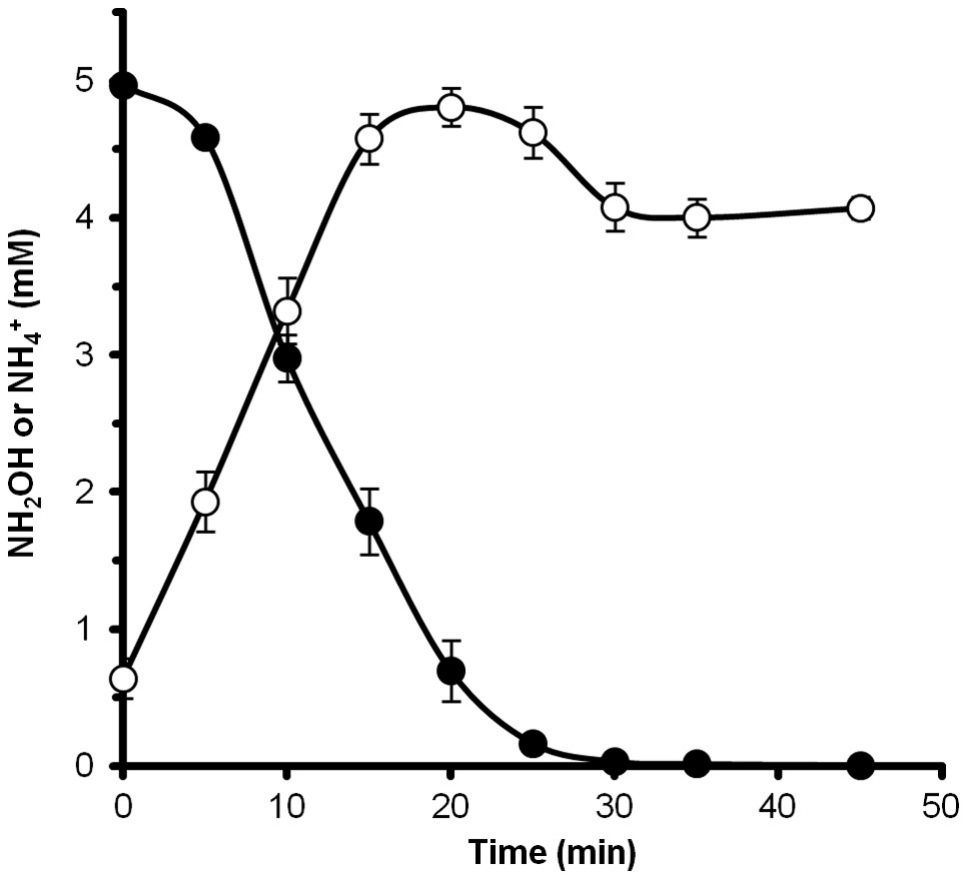
**Hydroxylamine is rapidly converted to ammonium by *N. profundicola***. The concentrations of hydroxylamine (closed circles) and ammonium (open circles) were followed after the addition of 5 mM hydroxylamine to a resting cell suspension. The points are the mean values from three replicates ± the standard deviation.

To determine if hydroxylamine could be metabolized by *N. profundicola*, washed suspensions of nitrate-grown cells were incubated in nitrate-free growth medium with hydrogen as the sole electron donor. Hydroxylamine was added as a potential electron acceptor and the concentrations of both hydroxylamine and ammonium were followed over time. The results of this experiment indicate that nitrate-grown cells of *N. profundicola* rapidly and completely convert hydroxylamine to ammonium (Figure 6). The specific rate of hydroxylamine conversion in this experiment was 28.6 μM min^−1^ 10^8^ cells^−1^. The ammonium production rate from nitrate calculated for exponential growth in Figure 3 (8–24 h) is ~3 μM min^−1^ (10^8^ cells)^−1^. Thus, the measured hydroxylamine conversion rate is far in excess of the ammonium production rate observed during growth of *N. profundicola* with nitrate. A similarly tight coupling between hydroxylamine production and removal was recently reported for the chemolithotrophic ammonia-oxidizing archaeon, *Nitrosopumilus maritimus*, where hydroxylamine oxidation is an essential catabolic step (Vajrala et al., 2013).

## Discussion

The data presented here are consistent with a new pathway for nitrate reduction in organisms that lack classical NO-forming (NirS or NirK) or ammonium-forming (NirB/NirA) nitrite reductases with *N. profundicola* strain AmH as a useful model system. A reverse-HURM pathway has been proposed whereby nitrite is reduced to hydroxylamine in the periplasm and subsequently reduced to ammonium in the cytoplasm (Campbell et al., 2009). The original pathway has been slightly modified here to include a more likely candidate for the proximal electron donor to Har/Hcp, a predicted 4Fe-4S containing protein encoded by NAMH_1302 (Figure 1). Homologs of NAMH_1302 are conserved in *C. concisus* (GenBank accession YP_001466207), *C. curvus* (YP_001408944), *C. fetus* (YP_892678), and *C. mediatlanticus* (ZP_01871213) that possess all other genes for the reverse-HURM pathway. In addition, NAMH_1302 displayed an increase in transcript abundance in *N. profundicola* cells grown on nitrate relative to ammonium.

While the production of hydroxylamine from nitrate and nitrite both via chemical (Rollefson and Oldershaw, 1932) and biological routes (Lindsey and Rhines, 1932) has long been known and a [HaoA]_3_ complex was shown to reduce nitrite to hydroxylamine when supplied with reductant (Kostera et al., 2010), the ratio of hydroxylamine vs. ammonium production from nitrite in the reverse-HURM pathway is not yet clear. The uptake of extracellular hydroxylamine by bacteria is known; exogenously supplied hydroxylamine accumulates readily in the anammoxosome of *Kuenenia stuttgartiensis* (Lindsay et al., 2001; Schmidt et al., 2004), a compartment that can be reached only by crossing both the cell and anammoxosome membranes. The proposed uptake of the highly mutagenic hydroxylamine necessitates protection against DNA lesions, which may partially explain the extensive complement of DNA repair systems identified in *N. profundicola* (Campbell et al., 2009). Hydroxylamine has also been proposed as an intermediate of ammonium production from nitrite in plants and algae as ferredoxin-dependent nitrite reductase displays high reactivity and specificity for this substrate (Kuznetsova et al., 2004; Hirasawa et al., 2010).

Unlike the quinone reductase function of HURM in ammonia-oxidizers (Klotz and Stein, 2008; Simon and Klotz, 2013), we propose that HURM in *N. profundicola* acts, similar to NrfAH, as a periplasmic quinol oxidase system that shuttles electrons from the quinol pool to reduce nitrite to hydroxylamine. It is likely that the reverse-HURM-pathway evolved from a common ancestor of the NrfAH and ONR ammonification module in anammox bacteria. We hypothesize that this occurred before HURM—including a covalently bound trimeric HaoA complex capable of hydroxylamine disproportionation—was used as a catabolic module in both anaerobic and aerobic ammonia-oxidizing bacteria (Jetten et al., 2009; Klotz and Stein, 2010; Campbell et al., 2011; Kern et al., 2011; Simon and Klotz, 2013). A similar reversal of function from reducing to oxidizing activity effected by covalent bond-directed complex formation has been discussed for the NO-reducing cytochrome *c*'-beta (*cytS*) and the NO-oxidizing cytochrome P460 (*cytL*) (Elmore et al., 2007). Genes encoding the NrfAH and reverse-HURM modules coexist in only one bacterial genome sequence, *C. fetus* 82-40 (Table 1). It was shown recently that octaheme cytochrome *c* hydroxylamine dehydrogenase evolved as a member of a multi-heme cytochrome *c* protein superfamily with functions in the nitrogen and sulfur cycles and that cytochrome *c*_M_552 (CycB), NrfH, and NapC are members of another cytochrome *c* superfamily (Bergmann et al., 2005; Klotz and Stein, 2008; Kern et al., 2011; Simon and Klotz, 2013). It is also known that NAP complexes in *Epsilonproteobacteria* generally do not contain NapC subunit proteins (Klotz et al., 2006; Sievert et al., 2008; Kern and Simon, 2009; Klotz and Stein, 2010; Simon and Klotz, 2013). The proposed reverse function of a [HaoA]_n_—*c*_M_552 (CycB/NapC) complex as a nitrite reductase—quinol oxidase complex is feasible given the presence of both transcripts and the absence of the critical tyrosine protein ligand in *N. profundicola* HaoA (Campbell et al., 2009), which separate the N-oxide reducing NrfA and ONR from the N-oxide oxidizing HAO complexes (Bergmann et al., 2005; Klotz and Stein, 2008; Kern et al., 2011; Simon and Klotz, 2013).

The ^15^N assimilation data established that *N. profundicola* incorporates nitrate-nitrogen into biomass during growth when it is provided as the sole nitrogen source. However, the vast excess production of ammonium from nitrate over the calculated demands for biosynthesis leads us to conclude that the primary function of nitrate reduction to ammonium by *N. profundicola* is that of an efficient respiratory electron acceptor. This conclusion is supported by the increased growth rates and yields in *N. profundicola* cultures with nitrate as the sole electron acceptor. Furthermore, the gene expression data agree with our proposal (Campbell et al., 2009) that this occurs via the concerted function of Nap, HURM ([HaoA]_3_ and *c*_M_552) and Har/Hcp as a novel N-assimilation and energy conservation pathway: the reverse-HURM pathway. Finally, the data indicate that *N. profundicola* has the ability to rapidly reduce hydroxylamine to ammonium, consistent with the proposal of free hydroxylamine as an intermediate in the reverse-HURM pathway. The high rate of hydroxylamine uptake observed likely explains why it is not observed consistently during growth of cultures on nitrate. Preliminary measurements of ammonium production from nitrate or nitrite in resting cells are similar to the 3 μM min^−1^ (10^8^ cells)^−1^ rate calculated from cultures, ~10-fold lower than the observed hydroxylamine consumption rate. Given these rates, hydroxylamine should be consumed immediately after production in cultures growing on nitrate. As hydroxylamine is mutagenic, we hypothesize that this reflects previous selective pressure to maintain low hydroxylamine levels during growth.

While labeling of biomass has been attempted with ^15^N-NH_2_OH in batch *N. profundicola* cultures, it has not yet been successful (data not shown). Given that hydroxylamine in batch culture must be supplied at low concentrations relative to the unlabeled nitrate to avoid toxicity and that the vast majority of N from hydroxylamine should be converted to ammonium (>90% based on Figure 3), this result is not surprising. Short-term labeling experiments with much higher concentrations of labeled hydroxylamine as the sole N-source may definitively establish this point.

In conclusion, the data presented here are consistent with a model where *N. profundicola* reduces nitrate via a free hydroxylamine intermediate. These experiments do not yet completely exclude the possibility that the reverse-HURM activity proposed in *N. profundicola* functions as an ammonium-forming nitrite reductase that transfers 6 electrons without releasing an N-oxide intermediate. Unpublished results indicate that a few point mutations in the *nrfA* gene can render pentaheme cytochrome *c* nitrite reductase into a leaky enzyme that releases N oxide intermediates and fails to convert nitrite stoichiometrically into ammonium (Jörg Simon, pers. communi.). Given the evolutionary relationship between *nrfA* and *haoA* (Bergmann et al., 2005; Simon and Klotz, 2013), the reverse-HURM module in *N. profundicola* might naturally function as an ammonium and hydroxylamine-producing enzyme complex whereas extant NrfA operates “leak-free” as an ammonium-producing nitrite reductase. Further experiments using inhibitors of Har/Hcp with nitrate or nitrite as a substrate in whole cells and/or biochemical studies of the reverse-HURM complex to determine the *in vitro* product should provide additional direct tests of this hypothesis. Irrespective of the specific mechanism, the extensive production of ammonium far in excess of biosynthetic needs by *N. profundicola* suggests that it and related *Epsilonproteobacteria* may serve an ecological role as a source of ammonium in nitrate rich, ammonium deprived, hypoxic environments where they are commonly found.

## Materials and methods

### Free energy calculations

Gibbs free energies (Table 2) were calculated from standard redox potentials of the half reactions by the Nernst equation (Δ G^0^ = −nFE^0^) or by the summation of half reaction free energies collected from the literature (Thauer et al., 1977; van der Star et al., 2008). All calculations were performed at standard conditions (1 M all species, 25°C, pH = 7).

### Culturing and nitrogen compound analysis

*N. profundicola* strain AmH (ATCC BAA-1463) was cultured and cells counted as previously described (Smith et al., 2008; Campbell et al., 2009). Ammonium in culture supernatants was quantified after derivatization with dansyl chloride and separation by HPLC with fluorescence detection (Shakila et al., 2001). Nitrate was quantified by HPLC using UV/Vis detection. Hydroxylamine concentrations were determined in culture filtrates (0.2 μm) as described by Frear and Burrell (1955).

### ^15^N labeling procedures

Stock solutions of 20 Atom% ^15^N-nitrate and ^15^N-ammonium were prepared by mixing >99 Atom% and natural abundance salts to prepare medium at a final concentration of 5 mM nitrate or ammonium as needed. Cells were harvested from ^15^N labeled cultures by centrifugation and the cell pellet washed three times with nanopure water to remove salts. The cell suspension was dried in tin capsules which were sent to the University of California-Davis stable isotope laboratory where ^15^N Atom % was determined by isotope ratio mass spectrometry. Control samples indicated that no isotopic contamination had occurred during sample processing (data not shown).

### Quantitative PCR

Gene-specific primers (Table 3) were used to quantify mRNA abundance in total RNA extracted from *N. profundicola* as previously described (Campbell et al., 2009).

**Table 3:** **Primers and conditions for Q-RT-PCR quantification of specific genes in total RNA samples extracted from *N. profundicola***.

**Gene**	**Primer name**	**Primer sequence (5^′^–3^′^)**	**RT step (^°^C, 3^′^)**	**Cycling conditions^1^**	**Efficiency**
*amtB*-1	0198_853F	TTGCAGGTCTTGTGGCTATTACT	42	95 (15″)–60 (1′)	0.85
	0198_1011R	GTGGATACCGAATGCATCTAATG			
*amtB*-2	amtB_397F	CCACAACCACAACACCTTTG	50	95 (15″)–58 (30″)–68 (15″)	0.81
	amtB_397R	GCGCTTATTATCGGTGCAAT			
*hao*	hao_915_F	TTGTCACCAAGCAGCTATCG	42	95 (15″)–60 (1′)	0.91
	hao_1116_R	TGGACCTTGAGGGTTACCAG			
*napA*	napA_F	CAAGCATATCCGCAGGAAGT	42	95 (15″)–58 (30″)–72 (15″)	0.93
	napA_R	CTGGACAATGGGATTCAACC			
*har*	har_656_F	GCAAATGGAACGGAACTTGT	50	95 (15″)–60 (1′)	0.90
	har_807_R	CGGATCGAAATTGTCGTTTT			
*napC*	napC_372F	CCACATGGTGAAAGAACCTG	42	95 (15″)–45 (15″)–72 (15″)	0.74
	napC_526R	GGTTTGGATGGGCAAAATTA			
NAMH_1302^2^	AmH_1302F2	TGTATGTAACGCAGGAGCCCTTA	42	95 (15″)−50 (30″)−72 (30″)	0.77
	AmH_1302R2	CATTATGCAGTGAAACGGTTCGT			
16S rRNA	AmH_16SF	AGGGAATTCCTGGTGTAGGG	42	95 (15″)–58 (30″)–72 (15″)	0.95
	AmH_16SR	TCGTTTAGGGCGTGGACTAC			

1 All PCRs included an initial 5′ denaturation at 95°C, 35 cycles of amplification, a 1′ 40°C step and a dissociation curve step.

2 Amplification of the AmH_1302 mRNA was done with an ABI 7500.

### Hydroxylamine uptake

*N. profundicola* was grown on H_2_ and formate as electron donors and nitrate as the sole electron acceptor to late exponential phase. Cells were harvested by centrifugation. All transfers and cell washings were performed in a Coy Laboratories anaerobic chamber. Cells were resuspended with nitrate-free growth medium to a final concentration of ~10^9^ cells ml^−1^ and incubated at the 45°C for 2 h under an atmosphere of 80% H_2_ + 20% CO_2_ prior to the addition of hydroxylamine (5 mM final concentration) from an anaerobically prepared stock solution. Aliquots of the cell suspension were filtered and assayed for hydroxylamine as described above and for ammonium by HPLC after derivatization with diethyl ethoxymethylenemalonate (Gómez-Alonso et al., 2007).

## Author contributions

Thomas E. Hanson, Barbara J. Campbell, and Martin G. Klotz collaboratively designed experiments and interpreted results, Thomas E. Hanson performed all nitrogen compound analyses and prepared samples for mass spectrometry, Barbara J. Campbell grew all cultures and performed quantitative PCR, Katie M. Kalis performed hydroxylamine uptake experiments, Mark A. Campbell identified NAMH_1302 as a relevant gene. Thomas E. Hanson drafted the paper, which was subsequently revised by Thomas E. Hanson, Barbara J. Campbell, and Martin G. Klotz.

## Conflict of interest statement

The authors declare that the research was conducted in the absence of any commercial or financial relationships that could be construed as a potential conflict of interest.
